# Recommendations for the follow-up care of female breast cancer survivors: a guideline of the Spanish Society of Medical Oncology (SEOM), Spanish Society of General Medicine (SEMERGEN), Spanish Society for Family and Community Medicine (SEMFYC), Spanish Society for General and Family Physicians (SEMG), Spanish Society of Obstetrics and Gynecology (SEGO), Spanish Society of Radiation Oncology (SEOR), Spanish Society of Senology and Breast Pathology (SESPM), and Spanish Society of Cardiology (SEC)

**DOI:** 10.1007/s12094-017-1801-4

**Published:** 2017-11-14

**Authors:** A. Barnadas, M. Algara, O. Cordoba, A. Casas, M. Gonzalez, M. Marzo, A. Montero, M. Muñoz, A. Ruiz, F. Santolaya, T. Fernandez

**Affiliations:** 1 Department of Medical Oncology, Hospital de la Santa Cruz y San Pablo, Barcelona, Spain; 2 Department of Radio-oncology, Parc de Salut Mar, Barcelona, Spain; 3 Department of Gynecology and Obstetrics, Hospital de la Vall d’Hebrón, Barcelona, Spain; 4 Department of Medical Oncology, Hospital Virgen del Rocío, Seville, Spain; 5 Montesa Healthcare Center, Servicio Madrileño de Salud, Madrid, Spain; 6 Catalonian Health Institute, Generalitat de Catalunya, Barcelona, Spain; 7 Department of Radio-oncology, Hospital Universitario HM Sanchinarro, Madrid, Spain; 8 Department of Medical Oncology, Hospital Clínic Barcelona, Barcelona, Spain; 9 Department of Medical Oncology, Instituto Valenciano de Oncología, Valencia, Spain; 10 Ciudad San Pablo Healthcare Center, Servicio Madrileño de Salud, Madrid, Spain; 11 Department of Cardiology, Hospital La Paz Madrid, Madrid, Spain

**Keywords:** Breast cancer, Treatment chronic side effects sequelae, Follow-up guidelines, Primary care, Specialized care

## Abstract

The increased incidence and decreased mortality of breast cancer have produced an increased number of breast cancer survivors. The type of sequelae and comorbidities that these patients present call for a collaborative follow-up by hospital-based specialized care and primary care. In this document, we present a guideline drafted and agreed among scientific societies whose members care for breast cancer survivors. The purpose of this guideline is to achieve the shared and coordinated follow-up of these patients by specialized care and primary care professionals. In it, we review the health issues derived from the treatments performed, with recommendations about the therapeutic approach to each of them, as well as a proposal for joint follow-up by primary and specialized care.

## Introduction

Breast cancer is the most common malignancy in the female population, with a cure rate that exceeds 80%. The gradual increase in incidence, associated with an aging population and the implementation of screening programs, together with lower mortality rates, account for an increased number of patients who are breast cancer survivors (BCSs) [1].

BCSs often have a life that is conditioned by the sequelae or morbidities related to treatment of the disease and, despite the high cure rates, many of the patients are no longer able to enjoy the quality of life achieved by women in our society. The treatments administered can leave physical, psychological, and psychosocial sequelae that may manifest or persist even years after having completed treatment and can interfere with their well-being and reincorporation into ordinary social and occupational activities [2].

Breast cancer is not a single disease, and treatment options depend on each tumor’s biology and behavior, as well as on each patient’s characteristics. Among patient characteristics, we must mention age, overall health status, and menopause status as being highly important factors when defining individualized treatment and preventing certain treatment-derived complications [3].

There are three essential aims that BCS follow-up pursues: (1) early diagnosis of relapses, (2) minimizing the impact of sequelae and complications derived from breast cancer diagnosis and treatment, and (3) encouraging healthcare and preventive measures to promote well-being and decrease risks to their health. Thus, a better quality of life might be achieved by helping BCSs to reintegrate as fully as possible into the various areas of their lives, i.e., family, work, and social activities [4, 5].

The need for specialized hospital-based care (SC) and primary care (PC) to share BCS patient follow-up activities has become evident in recent years and requires effective coordination. This shared, coordinated follow-up must ensure that the three previously named goals are met. The primary care physician plays a prominent role in detecting relapses and second neoplasms, dealing with late-onset effects of cancer treatment, managing comorbidity, psychological care, promoting healthy lifestyles, and recommending health prevention strategies, as well as normalizing the healthcare of patients who have been cured of breast cancer. Furthermore, BCSs continue to benefit from receiving care by the hospital-based specialist, especially during the first 5 years of follow-up [6–9].

In this document, we present a guideline that has been drafted and agreed among different scientific societies whose members care for BCSs. The purpose of this guideline is to archive that the follow-up of these patients will be shared, collaborative, and coordinated among SC and PC professionals.

## Treatment-derived health problems

As previously noted, the therapies administered may entail different sequelae that can translate into impaired quality of life for women who have overcome breast cancer. Some sequelae are late onset. Identifying these issues and correcting them can improve adaptation to daily life [9]. On the other hand, certain adverse effects may condition adherence to prolonged hormone therapy. It is also important to know whether the patients are receiving other medications or alternative therapies that might interfere with the activity of endocrine therapy. The PC physician is best suited to detect and control these side effects.

The side effects that call for special attention according to their frequency are as follows: lymphedema, effects derived from ovarian failure and menopause-related symptoms, overweight, cardiotoxicity, other vascular toxicities, neurotoxicity, ocular toxicity, cognitive impairment, skin alterations, and the risk of second neoplasms. The characteristics of these side effects and management recommendations are summarized in Table 1.

**Table 1:** Side effects and recommendations for their management

Side effect	Characteristics	Attitude/recommendation
Lymphedema	10–30% of patients who have undergone axillary node removal and 3–10% of patients treated with selective sentinel node biopsy [10] In recent years, the strategy of substituting axillary lymphadenectomy for axillary radiation is being assessed, without losing effectiveness and with a decreased risk of lymphedema [12]	Preventive measures (hygiene, hydratation, avoiding weights, and wounds) Rehabilitation In some cases, lymphedema surgery may be considered
Ovarian failure and menopause-related symptoms		
Repercussions of ovarian function	20 and 80% of women may present amenorrhea secondary to chemotherapy, which may be permanent. The risk depends on the schedule administered and patient’s age at the time of chemotherapy administration	Offer fertility-preserving methods Refer to Reproduction Services prior to systemic treatment administration [13] Do not recommend conception until 3–6 months after completing systemic treatment [13, 14] If the patient is receiving tamoxifen and wishes to become pregnant, hormone therapy must be discontinued between 3 and 6 months prior to allowing conception [13, 15]
Sexual activity	Decrease in libido or vaginal dryness are attributable to both the ovarian failure young women undergo after chemotherapy or hormone therapy and the side effects themselves of hormone therapy (gonadotropin analogues, SERM, aromatase inhibitors) The change in body image and local pain secondary to surgery can also cause dysfunction of sexual activity	Psychological support Use of vaginal lubricants The use of vaginal tablets or hormone creams is controversial
Hot flashes secondary to menopause	They are the result of induced menopause and are aggravated by hormone treatments Both tamoxifen and aromatase inhibitors (anastrozole, letrozole, exemestane) can cause or aggravate hot flashes	If severe, treatment with velafaxine or gabapentin can be used Acupuncture has demonstrated efficacy
Risk of endometrial disease	Women who receive tamoxifen for a long period have a higher risk of suffering endometrial cancer, although these neoplasms are general diagnosed very early have a good prognosis	In patients receiving tamoxifen, annual gynecological examinations are recommended. Postmenopausal women should be evaluated preferably by a gynecologist if they present vaginal bleeding
Bone health	Spontaneous or induced menopause (secondary to chemotherapy, gonadotropin analogues, or oophorectomy) involves decreased bone mineral density This effect can be relevant in young women with early menopause Hormone treatment, especially aromatase inhibitors in postmenopausal women produce a faster decline in bone mineral density with an increased risk of osteoporotic fractures	In menopausal patients, a baseline densitometry is recommended when starting endocrine therapy. Depending on the results, patients should be referred to a Bone Metabolism Service or follow ASCO/ESMO recommendations [16, 17]. Patients who are given aromatase inhibitors should receive calcium and vitamin D supplements. If osteoporosis is detected, add bone resorption inhibitors Recommend aerobic exercise Quit smoking
Joint pain	This is a very common side effect, particularly in patients treated with aromatase inhibitors	Increase frequency and duration of physical exercise Minor analgesics Acupuncture can be beneficial
Limited mobility of the scapulohumeral joint on the same side as the breast lesion	One side effect that can present long term following axillary radiation is decreased mobility of the scapulohumeral joint on the side that received radiation secondary to fibrosis in the pectoral muscle of the side affected [18]	Moderate, but constant physical exercise in the limb that received radiation On occasion, if improvement is not seen, the help of Rehabilitation Specialists should be requested
Overweight	Weight gain is common during treatment for breast cancer, especially in women in whom menopause is induced or who follow hormone deprivation treatment Furthermore, overweight has been recognized, not only as a risk factor for breast cancer but also as an unfavorable factor for relapse [19]	Monitor and control weight Low calorie diets Physical exercise (150 min/week) Psychological support
Cardiotoxicity and other vascular toxicities		Control concomitant diseases (hypertension, diabetes, obesity) Promotion of healthy lifestyles Refer to cardiology if signs of heart failure appear
	Yearly incidence of ventricular dysfunction of approximately 9% that exceeds 40% in patients over the age of 75 years or having prior heart disease	
	Toxicity due to anthracyclines is sometimes detected late and is more serious and often irreversible compared to toxicity due to trastuzumab [20], which tends to be reversible	
	Toxicity secondary to anthracyclines is unusual if cumulative doses of 250 mg/m^2^ of adriamycin or 550 mg/m^2^ of epirubicin [3, 21] are not exceeded	
	Cardiotoxicity due to trastuzumab usually appears during the active treatment phase and indicates that treatment must be withdrawn, although a high percentage of cases recover without sequelae. In women who have presented heart failure, there is no complete evidence as to whether it is possible to discontinue long-term cardiological treatment [22–24]	
	Deep-vein thrombosis (DVT) or pulmonary thromboembolism can be a side effect of tamoxifen and, less often, of aromatase inhibitors	If deep-vein thrombosis develops, refer patient to the oncologist to evaluate the advisability of continuing endocrine treatment
	In patients who have received complementary radiation therapy, especially in the case of tumors on the left side and when the internal mammary chain has been radiated, long-term follow up must be carried out, given the risk of late cardiac toxicity. Nevertheless, the more modern radiation techniques with three-dimensional planning and dose intensity modulation have made it possible to lower the incidence of this type of side effect [25, 26], although risk factors should be strictly controlled and the advisability of stress testing should be assessed [15]	
Neurotoxicity	This is a side effect associated with the administration of taxanes. Sensory neurotoxicity in the form of paresthesia and pain in the hands and feet causes great discomfort	Detect toxicity early There is no specific treatment Duloxetine, gabapentin, and pregabalin can improve symptoms
Ocular toxicity	Though uncommon, tamoxifen can increase the risk of cataracts	Refer to ophthalmologist if symptoms of blurry vision appear
Asthenia	Asthenia is a highly prevalent symptom in breast cancer that often persists after competing treatments [27] The use of a visual numeric scale is recommended to quantify the degree of asthenia and should be monitored	Rule out organic cause Psychoemotional support Physical activity
Cognitive impairment	Although there are few studies, many patients report memory loss or losing the ability to concentrate after chemotherapy that can last more than 20 years after treatment Methods are being investigated that enable us to diagnose and follow-up on this side effect	Assessment by Neurology Concentration and visual memory exercises Reading
Skin changes (dryness, alopecia, others)	Following treatment with chemotherapy, some women do not recover all their hair or present side effects of the skin and related structures, such as the nails	Evaluation by Dermatologist
Risk of second neoplasms	Patients who have undergone radiation therapy are at increased risk for a second neoplasm related to treatment with radiation. This phenomenon usually occurs many years after having received radiotherapy. The tumors that appear most often are neoplasms of the lung or angiosarcomas of the chest wall [28, 29]	Factors related to lung cancer should be avoided, especially with respect to smoking

In addition, there are general recommendations that must be followed [8, 9]:

- *Overweight avoidance* Weight gain has been associated with a worse treatment response and greater risk of relapse.
- *Healthy diet and exercise* Epidemiological studies have proven a benefit for patients following a low-fat diet together with at least 150 min of vigorous physical activity per week [10]. The recommended diet is high in fresh fruits, vegetables, and legumes (at least two pieces of fruit per day); patients are also recommended to lower their intake of red meat (to 1–2 times per week) and processed meats and increase consumption of blue fish, olive oil use and consume dairy products, as well as take advantage of all the elements comprising a Mediterranean diet.
- *Smoking avoidance*
- *Moderate alcohol intake* It is recommended that women abstain from drinking more than 20 g of alcohol per day. The following beverages equal 20 g of alcohol: 250 ml of beer, one glass of red wine (150 ml), or a quarter of a glass (25 ml) of a higher grade liquor (e.g., gin, whisky, anise, and tequila).
- *Use of complementary therapies* Some of these therapies could interfere with patient treatment. Acupuncture must be properly performed and with necessary aseptic measures to minimize the risk of infection. Complementary or integrative therapies cannot substitute for a specific antitumor treatment.
- *Awareness of symptoms that indicate possible relapse or second tumors* The following symptoms should be monitored: persistent bone pain that increases with movement and fails to remit with rest, persistent cough, dyspnea, asthenia, anorexia, or unexplained weight loss, vaginal bleeding in postmenopausal women, change in intestinal rhythm, rectal bleeding, or persistent headache or other neurological deficit. Thus, an early diagnosis can be made.
- *Confirmation of compliance with antiestrogenic hormone therapy* Control and positive reinforcement to maintain the prescribed treatment even over prolonged periods (5–10 years).
- *Return to work* Support and guidance appropriate to the patient’s stage of evolution. In the case of complementary treatment, it is recommended that patients wait for a reasonable time so that they can recover from the side effects of treatment.
- *Active alertness* of the appearance of symptoms suggestive of heart disease.

## Proposal of joint primary care and specialized care follow-up (Table 2)

Studies conducted thus far have revealed that radiological testing is not useful, except for yearly mammograms and that a PC physician or nurse can conduct follow-up if they are motivated and trained to care for the complications or morbidities that BCS present. Patient satisfaction will depend more on how they are treated than on where follow-up is undertaken. Studies have also concluded that relapses are detected similarly in PC or SC and that there are no differences in overall survival with any of the different follow-up schemes (with imaging studies and tumor markers compared with history, breast examination, and yearly mammogram), if the patient is guaranteed access to healthcare services in the presence of a symptom or sign of alarm [11].

**Table 2:** Proposed follow-up together with primary care

Risk group	Definition	Recommendation
Low risk	Hormone-sensitive tumor Size ≤ 2 cm (category pT1) No axillary metastases Low risk according to genomic platform Carcinoma “in situ”	Clinical check-up every 6 months, alternating with primary care (PC) for 5 years After 5 years, only PC will be responsible for check-ups Yearly mammogram
Intermediate risk	Hormone-sensitive tumor Size between 2 and 5 cm (category pT2) No nodal metastases or axillary metastases involving between 1 and 3 nodes Intermediate risk according to genomic platform	Check-up every 4 months together with PC for the first 2 years Every 6 months until the fifth year Only PC will be responsible for check-ups after that Yearly mammogram
High risk	Tumor not expressing hormone receptors Tumor with HER2 amplification High risk tumor according to genomic platform Tumor with metastases in more than 3 axillary nodes Tumor treated with neoadjuvant therapy Locally advanced tumor	Check-up every 4 months together with PC for 5 years After 5 years, every 6 months together with PC until 10 years Only PC will be responsible for check-ups after that Yearly mammogram

At every visit, the following should be performed: (i) Complete anamnesis evaluating the presence of comorbidities. (ii) Evaluation of hormone treatment compliance (whenever prescribed). (iii) Detection of side effects. (iv) Physical examination of the mammary glands, rib cage, or area of breast reconstruction and lymph node chain. (v) Foster a healthy lifestyle

However, long-term survivors who have overcome breast cancer require outstanding coordination and communication between PC and SC. It is proposed that patients already treated in the hospital be classified into three groups: (1) low risk; (2) intermediate risk, and (3) high risk. Table 2 summarizes the management recommendations coordinated between PC and SC.

Follow-up visits of female BCSs should include the following:

- History aimed at ruling out warning signs of relapse or the presence of sequelae, comorbidities, or second neoplasms.
- Adherence to adjuvant endocrine therapy when indicated.
- A physical examination that includes examination of the breasts and adjacent node regions.
- Bilateral, yearly mammograms in two projections, after conservative surgery of the breast or if the breast has been reconstructed. Annual follow-up visits will be maintained throughout their lifetime because these patients, unlike the healthy population who undergo biannual mammograms in screening campaigns, have a 5–10% probability of developing local relapse or a second neoplasm within 10 years post-treatment; this higher risk remains throughout their entire lives. Mammograms can be performed within the scope of PC or with the aid of the specialist in Obstetrics and Gynecology.
- Recommendations for prevention and health promotion.

### Role of the primary care physician

More and more women who have had breast cancer are being cared for in primary care. Optimal care by the PC physician includes the prevention, detection of relapses and possible second neoplasms, care for comorbidities, and the approach to late-onset effects of cancer treatment. It is also up to the PC physician to refer all women who have had breast cancer and present an accumulation of cases of cancer in the family to the reference Genetic Counseling Centers. The criteria for reference to a Genetic Counseling Center are as follows: having one or more first- or second-degree relatives with a breast or ovarian tumor under the age of 50; having a relative with bilateral breast tumors, having a male relative with breast cancer; having a relative with breast cancer with a triple-negative phenotype and less than 60 years of age [10]. Patients should also be referred to these centers when colon cancer is detected in different members of the family under the age of 50 at presentation.

Follow-up of BCS calls for a multidisciplinary approach and excellent coordination and communication between PC and SC. The oncologist should facilitate a case report when initiating and completing treatment; this report should include the type and clinical status of the tumor, treatment intent, treatments received, and toxicities that may have arisen during treatment. This information is fundamental to elaborating an individualized care plan that makes appropriate follow-up possible.

Below is the proposed minimum content that would be advisable to include in the report issued by SC to share patient information with PC:

### Initial oncological report

1. *Disease debut* (symptom duration, origin [population screening programs, fast track diagnostics, PC physician, others]).
2. *Diagnostic testing* (imaging studies performed to determine the diagnosis, pathology results of biopsy with immunohistochemical report, clinical TNM).
3. *Surgical procedure* (if performed, and postoperative TNM).
4. *Extension study, prognostic and predictive factors, results of the genomic study of the risk of relapse* (if performed).
5. *General treatment approach and anticipated date of initiation* Medical Oncology’s treatment prescription:
  - Drugs
  - Sequence of administration. Courses
  - Anticipated duration.
6. Radiation treatment approach (when applicable):
  - Volumes to be treated, total dose, and fractioning.
  - Radiotherapy technique: 3D, IMRT, partial radiation, intraoperative radiation, source, and energy.
  - *Prevention guidelines* considering possible side effects of treatment.
  - Report if patient is participating in a Clinical Trial.
  - *Estimation of time off work* Consideration must be paid as to whether the time off work has to do with the characteristics of the workplace, associated risks, and compatibility with the degree of functioning following treatment.
  - Date of the next visit to SC. In some Autonomous Communities, the PC physician can consult them, but not in all of them.

### Report upon completion of the initial phase of treatment

Once the initial stage of treatment is completed (surgery, treatment with chemotherapy, and/or targeted drugs and radiotherapy), after the first 6–15 months, a new stage opens. This period is no less important than the diagnostic stage, given the uncertainty surrounding the quality and continuity of life, during which information about the patient’s status must be shared again with PC physicians.

1. Regarding clinical situation during the initial stage of treatment.
  - Full report that includes the previously listed items (points 1, 2, 3, and 4);
  - All treatments received: radiotherapy, hormone therapy, or biological therapy, and if the patient has been included in a clinical trial. Treatment duration and related side effects and their treatment prevention (points 5 and 6);
  - Information about the risk of cardiotoxicity and type of cardiovascular monitoring undertaken;
  - Overall evolution;
  - Information regarding schedules for follow-up: tests and timing, outline of visits with SC.
2. Regarding clinical recommendations
  - Warning signs that a new appointment should be set up with SC;
  - Specific approach to lymphedema: when, how, and who to refer to;
  - Motor rehabilitation if necessary and recommendations regarding physical exercise;
  - Specific approach toward side effects of hormone medication. Recommendations for the prevention/treatment of osteoporosis and related side effects;
  - Control of mammary implants/expander/prosthetic replacement, and related events;
  - Dietary recommendations;
  - Special considerations regarding patients’ health issues and comorbidities, and
  - Complementary therapies and their repercussions.
3. Psychosocial and occupational recommendations
  - Specific aspects to be considered depending on the patient’s psychological situation, family and social impact. Need for specific social support;
  - Psychological approach. Fear of relapse;
  - Recommendations about sexuality and contraception;
  - Recommendations about returning to work or resuming previous activities. Pursuing a life path/professional plan, and
  - In certain women who want to be mothers, recommendations will be made about the best time to conceive. Young women who have preserved oocytes or ovarian cortex should be referred to Fertility Services at the Obstetrics and Gynecology Department.
